# Changes in the Expression Profile of Growth-Associated Protein 43 in Degenerative Lumbosacral Stenosis

**DOI:** 10.3390/jcm14041223

**Published:** 2025-02-13

**Authors:** Dawid Sobański, Małgorzata Sobańska, Rafał Staszkiewicz, Damian Strojny, Beniamin Oskar Grabarek

**Affiliations:** 1 Department of Neurosurgery, Szpital sw. Rafala in Cracow, 30-693 Cracow, Poland; sobanskamalgorzata05@gmail.com; 2 Collegium Medicum, WSB University, 41-300 Dabrowa Gornicza, Poland; rafalstaszkiewicz830@gmail.com (R.S.); strojny.ds@gmail.com (D.S.); bgrabarek7@gmail.com (B.O.G.); 3 Department of Neurosurgery, 5th Military Clinical Hospital with the SP ZOZ Polyclinic in Krakow, 30-901 Krakow, Poland; 4 Department of Neurosurgery, Faculty of Medicine in Zabrze, Academy of Silesia, 40-555 Katowice, Poland; 5 Institute of Health Care, National Academy of Applied Sciences in Przemyśl, 37-700 Przemyśl, Poland; 6 New Medical Techniques Specialist Hospital of St. Family in Rudna Mała, 36-060 Rzeszów, Poland

**Keywords:** degenerative spinal stenosis, growth-associated protein 43 (GAP-43), pain biomarkers, neuroplasticity, lifestyle factors

## Abstract

**Background:** Degenerative spinal stenosis is a common condition associated with structural degeneration and pain, yet its molecular underpinnings remain incompletely understood. Growth-associated protein 43 (GAP-43), a key player in neuronal plasticity and regeneration, may serve as a biomarker for disease progression and pain severity. This study investigates the expression of GAP-43 at the mRNA and protein levels in the ligamentum flavum of affected patients. **Methods:** Samples were collected from 96 patients with degenerative spinal stenosis and 85 controls. *GAP-43* mRNA expression was analyzed using reverse transcription–quantitative polymerase chain reaction (RT-qPCR), while protein levels were quantified via enzyme-linked immunosorbent assay (ELISA) and Western blot. Pain severity was assessed using the visual analog scale (VAS), and associations with lifestyle factors were analyzed. **Results:** *GAP-43* mRNA expression was significantly downregulated in the study group compared to the controls (fold change = 0.58 ± 0.12, *p* < 0.05), with an inverse correlation to VAS pain severity (fold change = 0.76 at VAS 4 vs. 0.36 at VAS 10). Conversely, GAP-43 protein levels were markedly elevated in the study group (5.57 ± 0.21 ng/mL) when compared to controls (0.54 ± 0.87 ng/mL, *p* < 0.0001). Protein levels were also correlated with lifestyle factors, including smoking and alcohol consumption (*p* < 0.05). **Conclusions:** GAP-43 shows potential as a biomarker for pain severity and disease progression in degenerative spinal stenosis, in a manner influenced by lifestyle factors. Further research is needed to explore its diagnostic and therapeutic applications.

## 1. Introduction

Lumbosacral (L/S) spine stenosis is a condition characterized by the narrowing of the spinal canal, leading to the compression of neural and vascular structures within the lumbar region. Clinically, this condition presents with pain in the buttocks and lower limbs, accompanied by lower back pain in some cases [1,2]. A hallmark symptom of lumbar stenosis is neurogenic claudication, which manifests as pain or discomfort triggered by standing and walking, and can be alleviated by sitting, lying down, or adopting a forward-bending posture [3]. This condition predominantly affects the L4/L5 vertebral level, followed by L3/L4 and L2/L3, with less frequent involvement of the L5/S1 and L1/L2 segments [4]. Patients with central or lateral recess stenosis may experience persistent pain, including during rest, at night-time, or when sneezing. In cases where neurological symptoms are absent or minimal, conservative treatment, including rehabilitation and analgesic medication, is often effective [5,6,7]. However, surgical intervention becomes necessary for individuals with progressive neurological deficits, increasing disability, or refractory pain. Surgical intervention for lumbar stenosis primarily aims to relieve neural element compression, with decompressive laminectomy being one of the most widely used techniques [8]. This procedure involves the removal of the vertebral arch, excision of the yellow ligament, trimming of the joint, and widening of the nerve root canal to achieve optimal decompression [8]. While the clinical manifestations of degenerative lumbar spine stenosis, such as pain and functional impairment, are well documented, emerging evidence highlights an underlying molecular basis for these symptoms [9,10]. Neurotrophic factors have garnered attention for their roles as key mediators in pain generation, modulation, and nerve repair processes [11]. These molecules act as neurotransmitters and modulators of neuronal plasticity, offering insights into the mechanisms linking structural degeneration to symptomatic expression. Investigating the molecular dynamics within affected tissues, such as the ligamentum flavum, may uncover novel therapeutic targets for managing lumbar stenosis and its associated pain syndromes [12].

In this context, proteins involved in neuronal plasticity, such as growth-associated protein 43 (GAP-43), may also play a significant role. GAP-43, also known as neuromodulin, is critical for neuronal development, regeneration, and intracellular signaling. Initially identified in synaptosomal plasma membranes, it has been recognized for its functions in axonal growth, cytoskeletal remodeling, and neuroplasticity [13,14]. Beyond its traditional association with neuronal tissues, recent findings have revealed its expression in skeletal muscle, where it regulates intracellular calcium dynamics, suggesting broader physiological relevance [15].

While the role of GAP-43 in neural and muscular tissues is well documented, its presence and potential functions in the ligamentum flavum remain unexplored. GAP-43, often referred to as a “growth cone protein”, is essential for axonal growth, synaptic plasticity, and intracellular signaling, particularly in response to injury and regeneration. In neural tissues, GAP-43 is a key regulator of neuroplasticity, modulating cytoskeletal remodeling and intracellular calcium dynamics. Interestingly, recent studies have also demonstrated its expression in skeletal muscle, where it influences calcium handling and contractile properties, suggesting a broader physiological role beyond the nervous system. However, the function of GAP-43 in ligamentous tissues, especially the ligamentum flavum, is currently unknown [16,17]. Given the established role of GAP-43 in modulating cellular plasticity and the response to mechanical stress in other tissues, it is plausible that it could influence similar processes in the ligamentum flavum. For instance, GAP-43 could be involved in mediating the cellular response to chronic mechanical stress, inflammation, and micro-injury within the ligament, contributing to tissue thickening and fibrosis [18,19].

Recent evidence suggests that GAP-43 is involved in the response to neural root damage and rootlet injury caused by ischemia, a process that could contribute to pain and functional impairment in spinal pathologies [20,21]. Neural root ischemia, which is commonly observed in degenerative spinal stenosis, results in reduced blood flow and oxygenation, leading to nerve root injury and the activation of neuroplastic responses [10,22]. In this context, GAP-43 plays a pivotal role in axonal regeneration and synaptic remodeling, particularly under conditions of injury. While this mechanism is well established in the central and peripheral nervous systems, its role in the ligamentum flavum and its potential correlation with ischemic nerve root damage remains under-explored. Despite this plausible hypothesis, no studies have investigated the expression or functional role of GAP-43 in the ligamentum flavum to date. This represents a significant gap in the current understanding of spinal degeneration at the molecular level. Identifying the role of GAP-43 in this context could reveal new insights into the mechanisms underlying ligament hypertrophy and spinal stenosis. Furthermore, it could open new avenues for therapeutic interventions targeting molecular pathways involved in pain generation, tissue remodeling, and ligament degeneration. The objective of this study was to investigate how GAP-43 concentrations in the yellow ligamentum flavum of the L/S region vary in association with the degree of degenerative changes, intensity of pain, lifestyle patterns, behavioral habits, and the presence of comorbid conditions.

## 2. Materials and Methods

### 2.1. Study Group Characteristics

This study builds upon the findings and methodologies established in our prior research [23,24].

The study cohort comprised 96 patients, including 46 women (48%) and 50 men (52%), with a mean age of 68.3 ± 2.4 years. These patients were scheduled to undergo extended fenestration and foraminotomy surgeries. The diagnosis of spinal stenosis in the lumbosacral (L/S) region was confirmed using clinical evaluations, physical assessments, and magnetic resonance imaging (MRI) with slice thicknesses of 3 mm and 4 mm across various planes. Participants provided self-reported information regarding smoking and alcohol use; however, specific details about duration and quantity were not recorded. Eligibility criteria for inclusion in the study required documented degenerative stenosis of the L/S spine on imaging, an age range of 18 to 80 years, no significant contraindications for surgical or internal medical procedures, and a history of unsuccessful conservative treatment lasting at least six months. The participants needed to refrain from anticoagulants or discontinue them as advised, have no history of hormonal or gastrointestinal disorders, and not be pregnant or lactating. The exclusion criteria involved an absence of degenerative stenosis in imaging studies, a history of prior spinal surgery at the L/S level, effective conservative treatment, or contraindications to surgery. Other exclusions included individuals with unbalanced hormonal disorders, gastrointestinal issues such as malabsorption, or those who took vitamin or mineral supplements registered as medications in the past six months. Patient comorbidities, including diabetes, thyroid disorders, metabolic disorders, and heart diseases, were documented as part of the study group characteristics due to their potential influence on GAP-43 expression levels. These conditions, which are known to contribute to chronic inflammation, oxidative stress, and altered neuroplasticity, may impact the molecular pathways involved in degenerative spinal diseases. The presence of such comorbidities was recorded through medical history and patient interviews. However, these factors were not independently controlled in this study, which could introduce variability in GAP-43 expression patterns.

### 2.2. Pain Assessment

This study builds upon the findings and methodologies established in our prior research [23,24].

Pain intensity was measured using a 10-point visual analog scale (VAS), with 0 representing the absence of pain and 10 signifying the highest level of pain imaginable. No participants reported pain levels from 0 to 3. Pain levels among participants were distributed as follows: 19 reported level 4, 22 reported level 5, 23 reported level 6, 9 reported level 7, 8 reported levels 8 or 9, and 7 reported level 10.

### 2.3. Neurosurgical Procedure

This study builds upon the findings and methodologies established in our prior research [23,24].

Extended fenestration and foraminotomy were conducted under general anesthesia. After a skin incision and paraspinal muscle dissection, the hypertrophied ligamentum flavum was excised with Kerrison bone biters. Decompression of the dural sac and nerve roots was completed, followed by saline irrigation and incision closure. An operating microscope was used throughout. Patients without complications were discharged on the third day and attended a follow-up four weeks postoperatively.

### 2.4. Control Group Characteristics

This study builds upon the findings and methodologies established in our prior research [23,24].

The control group included 85 individuals, consisting of 39 women (46%) and 46 men (54%), with a mean age of 49.17 ± 2.65 years. Tissue samples were obtained postmortem during forensic autopsies or organ donation processes. Smoking, alcohol use, and diabetes status were documented, but no additional details on duration or quantity were available. Hematoxylin and eosin (H&E) staining confirmed the absence of degenerative changes in the ligamentum flavum. Two independent neurosurgery specialists verified sample eligibility. Inclusion criteria for the control group included informed consent, age between 18 and 80 years, no history of neoplastic diseases, the absence of degenerative spine disease, and no history of traumatic spine injuries. Participants also needed to have no hormonal or gastrointestinal disorders and should not have taken supplements registered as medications within the past six months. Exclusion criteria included the presence or history of degenerative spine disease, traumatic injuries to the spine, or neoplastic conditions. Additional exclusions mirrored the criteria for the study group, including pregnancy, lactation, and use of certain medications.

### 2.5. Preparation and Analysis of Collected Material for Molecular Testing

This study builds upon the findings and methodologies established in our prior research [23,24].

Ligamentum flavum samples were thoroughly washed and stored in sterile Eppendorf tubes with RNAlater reagent (Invitrogen Life Technologies, Carlsbad, CA, USA) to preserve RNA integrity. These samples were kept at −80 °C until molecular analyses, including mRNA analysis, ELISA, and a Western blot, were performed. Tissue samples were obtained exclusively from the ligamentum flavum, and care was taken to avoid contamination with annulus fibrosus or other adjacent tissues. Sample collection was guided by intraoperative anatomical landmarks and verified by two independent neurosurgeons (Dawid Sobański and Rafał Staszkiewicz).

### 2.6. RNA Extraction and Quality Assessment

This study builds upon the findings and methodologies established in our prior research [23,24]. Firstly, the obtained samples were minced using a hand-held homogenizer (T18 Digital Ultra-Turrax, IKA Polska Sp. z o.o., Warsaw, Poland) until no solid fragments remained. Then, RNA was extracted using TRIzol reagent (Invitrogen Life Technologies, Carlsbad, CA, USA). Tissue homogenization was conducted with a T18 Digital Ultra-Turrax device (IKA Polska, Warsaw, Poland). The isolates were treated with DNase I and processed using the RNeasy Mini Kit (Qiagen, Valencia, CA, USA), then dried and stored at −80 °C. RNA quality and concentration were evaluated via agarose gel electrophoresis with ethidium bromide staining and a Nanodrop spectrophotometer (Thermo Fisher Scientific, Waltham, MA, USA). High-quality RNA was indicated by clear 28S and 18S rRNA bands and purity ratios of 1.80–2.00.

### 2.7. Reverse Transcription–Quantitative Polymerase Chain Reaction (RT-qPCR)

This study builds upon the findings and methodologies established in our prior research [23,24].

RT-qPCR was performed using a 50 µL reaction mixture with reverse transcription at 45 °C for 10 min, polymerase activation at 95 °C for 2 min, and 40 cycles of denaturation (95 °C for 5 s), hybridization (60 °C for 10 s), and annealing (72 °C for 5 s). GAP-43 mRNA levels were assessed using specific primers: forward: 5′-GGCCGCAACCAAAATTCAGG-3′; reverse: 5′-CGGCAGTAGTGGTGCCTTC-3′. Beta-actin (ACTB: forward: 5′-TCACCCACACTGTGCCCATCTACGA-3′; reverse 5′-CAGCGGAACCGCTCATTGCCAATGG-3′) and 18S rRNA (forward: 5′-CGGACAGGATTGACAGATTGA-3′, reverse: 5′-GCCAGAGTCTCGTTCGTTAT-3′) served as endogenous controls. Sensi-Fast One-Step Probe Assay reagents (Bioline, London, UK) were used, and relative GAP-43 expression was calculated with the 2^–∆∆Ct^ method, where values > 1 indicated upregulation and values < 1 indicated downregulation compared to controls.

### 2.8. ELISA Test

This study builds on methodologies from previous research [23,24]. Ligamentum flavum samples were minced using a hand-held homogenizer (T18 Digital Ultra-Turrax, IKA Polska Sp. z o. o., Warsaw, Poland) until no solid fragments remained, and incubated for 12 h at 4 °C in a lysis buffer containing guanidine hydrochloride, sodium acetate, triton, and protease inhibitors (Sigma-Aldrich, St. Louis, MO, USA). After centrifugation, the supernatants were stored at −20 °C. ELISA was conducted with a polyclonal anti-GAP-43 antibody (STI, Poznan, Poland; catalog number bs-0154R; dilution 1:500) following the manufacturer’s protocol. Positive controls used human HeLa cells, and negative controls omitted the primary antibody. Each assay was performed in triplicate, and mean values were analyzed. Detailed ELISA and Western blot protocols have been previously reported [23,24].

### 2.9. Western Blot Analysis

Western blotting was performed following methods described in prior studies [23,24]. Ligamentum flavum samples were washed with PBS and then minced using a hand-held homogenizer (T18 Digital Ultra-Turrax, IKA Polska Sp. z o.o., Warsaw, Poland) until no solid fragments remained. The homogenized samples were then processed in a RIPA buffer containing protease and phosphatase inhibitors (Sigma-Aldrich, St. Louis, MO, USA). Homogenates were centrifuged, and the supernatants were stored at −80 °C. Protein concentrations (20–100 µg) were measured using a BCA assay (Thermo Fisher Scientific, Waltham, MA, USA). Proteins were separated via SDS-PAGE, transferred to PVDF membranes, and probed with a polyclonal anti-GAP-43 antibody (STI, Poznan, Poland; catalog number bs-0154R; dilution 1:200). Beta-actin (Santa Cruz Biotech, Dallas, TX, USA) served as the control, and detection utilized an HRP-conjugated secondary antibody (BioRad, Milan, Italy).

### 2.10. Statistical Analysis

Statistical analysis was performed using Statplus software (AnalystSoft Inc., Brandon, FL, USA, https://www.analystsoft.com/en/products/statplusmacle/), with a significance threshold of *p* < 0.05. Data normality was tested with the Shapiro–Wilk test. Group comparisons were analyzed using one-way ANOVA with Scheffe’s post hoc test, and pairwise comparisons were conducted with Student’s *t*-test. Linear regression models evaluated the relationships between VAS scores, lifestyle factors, and GAP-43 levels, while multivariate regression accounted for the combined effects. Interaction terms and multicollinearity were assessed to validate the models, with separate analyses performed for the study and control groups to examine differential effects.

## 3. Results

### 3.1. Transcriptional Activity of the mRNA GAP-43 in the Control and Test Samples

**Figure 1:**
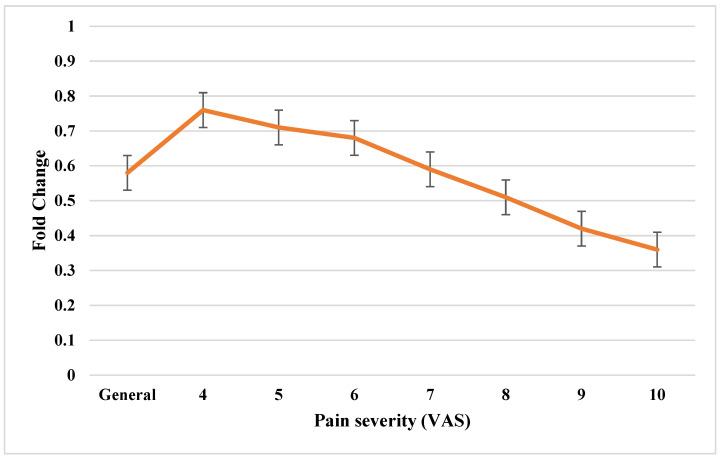
Changes in expression pattern of *GAP-43* mRNA in the yellow ligamentum flavum obtained from the test group, including pain severity (RT-qPCR).

*GAP-43* mRNA expression was significantly downregulated in the study samples compared to the control group (FC = 0.58 ± 0.12; *p* < 0.05). Further analysis investigated whether *GAP-43* transcriptional activity was associated with the severity of pain symptoms (scale 4–10). The results revealed a negative correlation between *GAP-43* mRNA expression and pain severity, with fold changes (FC) decreasing progressively from 0.76 at pain severity 4 to 0.36 at severity 10. ANOVA confirmed that the differences in *GAP-43* transcriptional activity across pain severity levels were statistically significant (*p* < 0.05). These findings, illustrating the inverse relationship between *GAP-43* mRNA transcriptional activity and pain severity, are presented in Figure 1.

### 3.2. Alterations in GAP-43 Protein Levels Assessed by ELISA

ELISA results revealed a significant reduction in GAP-43 protein levels in the study group compared to the control group. The GAP-43 concentration in the control group was 5.57 ± 0.21 ng/mL, while the study group exhibited a markedly lower concentration of 0.54 ± 0.87 ng/mL (*p* < 0.05). Additionally, GAP-43 levels showed a positive correlation with pain severity, as measured by the visual analog scale (VAS), with statistically significant results (*p* < 0.05). Detailed results and statistical analyses are summarized in Table 1.

Data are presented as mean ± standard deviation, as follows: 1, Statistically significant differences in GAP-43 expression between the study and control groups (Student’s *t*-test); 2, significant differences between GAP-43 expression at VAS 4 and VAS 6 (Scheffe’s post hoc); 3, significant differences between GAP-43 expression at VAS 4 and VAS 7 (Scheffe’s post hoc); 4, significant differences between GAP-43 expression at VAS 4 and VAS 8 (Scheffe’s post hoc); 5, significant differences between GAP-43 expression at VAS 4 and VAS 9 (Scheffe’s post hoc); 6, significant differences between GAP-43 expression at VAS 4 and VAS 10 (Scheffe’s post hoc); 7, significant differences between GAP-43 expression at VAS 5 and VAS 7 (Scheffe’s post hoc); 8, significant differences between GAP-43 expression at VAS 5 and VAS 8 (Scheffe’s post hoc); 9, significant differences between GAP-43 expression at VAS 5 and VAS 9 (Scheffe’s post hoc); 10, significant differences between GAP-43 expression at VAS 5 and VAS 10 (Scheffe’s post hoc).

### 3.3. Protein Expression Levels of GAP-43 in Samples Analyzed via Western Blot

**Figure 2:**
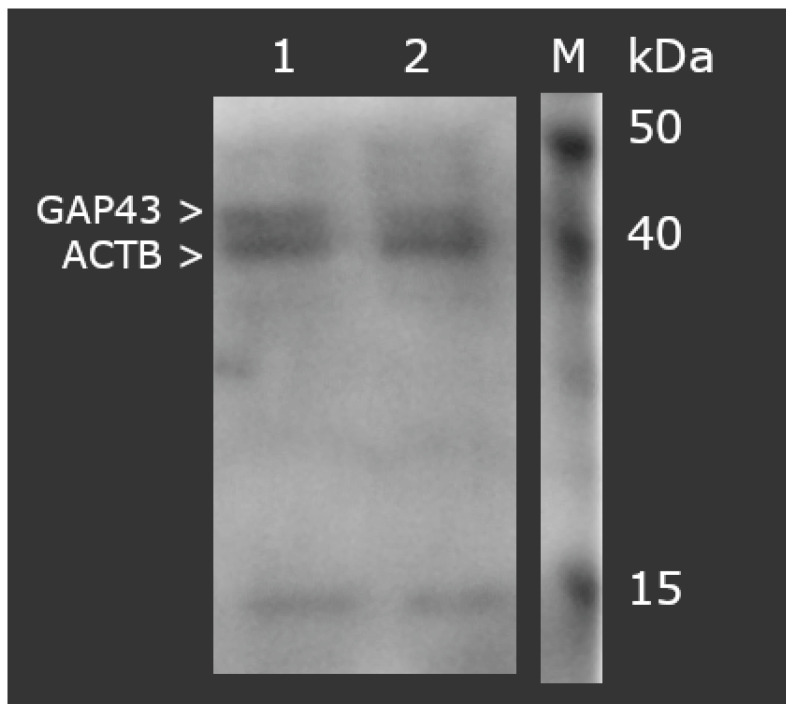
Normalized GAP-43 expression in ligamentum flavum samples, adjusted for ACTB levels (Western blot analysis). GAP-43: growth-associated protein 43; ACTB: beta-actin; M: molecular weight marker; lane 1: study group; lane 2: control group.

The Western blot analysis of GAP-43 protein expression in degenerated and control ligamentum flavum samples corroborated the ELISA findings. Figure 2 presents a representative electropherogram, demonstrating reaction specificity and sample integrity, confirmed by the ACTB band (molecular weight 42 kDa). The normalized optical density of GAP-43 (molecular weight 43 kDa) relative to ACTB was significantly elevated in the study group (0.48 ± 0.56) compared to the control group (0.22 ± 0.06, *p* < 0.05).

### 3.4. Impact of Lifestyle Factors on GAP-43 Expression at mRNA and Protein Levels in Degenerated Ligamentum Flavum Samples

*GAP-43* mRNA expression was generally downregulated in the study group compared to the control group across demographic and lifestyle factors (Table 2; *p* < 0.05). Females had slightly higher expression than males in both groups, but the difference was not statistically significant (Table 2; *p* > 0.05).

Across BMI categories, the control group consistently showed higher mRNA levels, with normal-weight individuals having the lowest expression and overweight or obese participants exhibiting progressively higher levels, suggesting that BMI may influence GAP-43 transcription (Table 2; *p* < 0.05).

Diabetic individuals had marginally higher mRNA expression than non-diabetics in both groups, but this was not significant (Table 2; *p* > 0.05). In the control group, smokers and non-smokers exhibited higher expression levels than those in the study group, whereas smokers showed notably reduced transcription (Table 2; *p* > 0.05). Alcohol consumption was linked to lower mRNA expression in the study group, with non-drinkers showing higher levels, indicating the possible regulatory role of lifestyle factors (Table 2; *p* < 0.05).

GAP-43 protein levels were significantly upregulated in the study group across all variables compared to the control group (Table 3; *p* < 0.05). Females and males in the study group exhibited higher protein levels than their control counterparts, indicating that GAP-43 upregulation is a general response to degenerative changes, regardless of gender (Table 3; *p* > 0.05).

Protein expression increased with higher BMI categories in both groups, with overweight and obese participants in the study group showing the highest levels (Table 3; *p* > 0.05). Diabetic individuals and smokers in the study group displayed significantly elevated GAP-43 levels compared to non-diabetic participants and non-smokers, respectively (Table 3; *p* < 0.05). Alcohol consumption was also associated with increased GAP-43 expression in the study group, suggesting a role for lifestyle factors in regulating GAP-43 in ligamentum flavum tissue (Table 3; *p* < 0.05).

### 3.5. Regression Analysis of Factors Potentially Influencing GAP-43 Levels in Ligamentum Flavum

#### 3.5.1. Univariate Regression Analysis of Factors Influencing GAP-43 Levels in Control and Study Groups

Table 4 presents the results of univariate regression analyses, showing the relationships between various demographic and lifestyle factors and GAP-43 expression levels in the control and study groups. Both mRNA and protein expression levels were analyzed, with the correlation coefficients (r) and coefficients of determination (R^2^) provided for each variable. Gender was found to have a weak correlation with GAP-43 expression levels in both groups. The mRNA expression showed a slightly higher correlation in the study group (r = 0.25, R^2^ = 0.06) when compared to the control group (r = 0.19, R^2^ = 0.36). For protein expression, the correlations were similar across both groups, with slightly higher values observed in the study group (r = 0.28, R^2^ = 0.08) compared to the control group (r = 0.22, R^2^ = 0.05). BMI showed a stronger correlation with GAP-43 expression levels, particularly in the study group. The mRNA expression in the study group had a correlation coefficient of r = 0.68 and R^2^ = 0.46, indicating a moderate relationship. Protein expression showed an even stronger correlation, with r = 0.81 and R^2^ = 0.66. In the control group, the correlation was also substantial for protein expression (r = 0.68, R^2^ = 0.46), suggesting that BMI is an important factor influencing GAP-43 levels. Diabetes presented moderate correlations with both mRNA and protein expression levels. In the study group, the mRNA expression showed a correlation coefficient of r = 0.76 and R^2^ = 0.58, while the protein expression had r = 0.72 and R^2^ = 0.52. The control group exhibited slightly weaker correlations. Smoking demonstrated a strong positive correlation with GAP-43 expression levels, particularly in the study group. The mRNA expression showed a correlation coefficient of r = 0.85 and R^2^ = 0.72, and protein expression reached r = 0.89 and R^2^ = 0.79. In the control group, the correlations were weaker, with protein expression showing r = 0.80 and R^2^ = 0.64. Alcohol consumption exhibited the highest correlations with GAP-43 expression levels. In the study group, the mRNA expression had r = 0.91 and R^2^ = 0.83, while protein expression reached r = 0.96 and R^2^ = 0.92. The control group showed similarly high correlations, indicating that alcohol consumption is a significant factor influencing GAP-43 levels in both groups.

#### 3.5.2. Multivariate Regression Analysis of Factors Influencing GAP-43 Levels in Ligamentum Flavum: Control and Study Groups

Table 5 summarizes the multivariate regression analyses, illustrating the combined effects of demographic and lifestyle factors on GAP-43 mRNA and protein expression in the control and study groups. Coefficients and *p*-values reflect the strength and significance of each variable’s influence.

Gender did not significantly affect GAP-43 expression levels, as indicated by *p*-values > 0.05 for both mRNA and protein expression across groups.

BMI emerged as a significant predictor in the study group, with coefficients of 0.401 for mRNA (*p* < 0.0001) and 0.385 for protein expression (*p* < 0.0001). In the control group, BMI also influenced protein expression (coefficient = 0.29; *p* = 0.08).

Diabetes significantly predicted GAP-43 protein levels in the study group (coefficient = 0.495; *p* = 0.029). In the control group, diabetes was also a significant predictor of protein expression (coefficient = 0.25; *p* < 0.0001).

Smoking significantly impacted GAP-43 levels in the study group, with coefficients of 0.332 (*p* = 0.018) for mRNA and 0.322 (*p* = 0.015) for protein expression. In the control group, smoking strongly influenced protein expression (coefficient = 0.37; *p* < 0.0001).

Alcohol consumption was the most robust predictor of GAP-43 levels in both groups. In the study group, alcohol consumption showed coefficients of 0.345 (*p* < 0.0001) for mRNA and 0.278 (*p* < 0.0001) for protein expression. Similar trends were observed in the control group, where alcohol consumption significantly affected both mRNA and protein expression levels.

In summary, the univariate regression analysis in Table 4 highlighted individual correlations between GAP-43 expression levels and various factors, whereas the multivariate regression analysis in Table 5 demonstrated the combined effects of these variables. BMI, diabetes, smoking, and alcohol consumption were identified as significant predictors of GAP-43 levels, particularly in the study group, suggesting that these factors may contribute to the molecular changes observed in degenerative yellow ligamentum flavum.

#### 3.5.3. Multivariate Analysis of GAP-43 Protein Levels: Impact of Pain Severity and Lifestyle Factors

In turn, Table 6 summarizes the multivariate regression analysis examining the relationships between GAP-43 protein levels and various factors, including pain severity and lifestyle behaviors. The VAS pain score showed a strong positive association with GAP-43 protein levels, with a significant coefficient of 3.12 and a *p*-value less than 0.001 in the multivariate model. While BMI demonstrated a significant positive association in the univariate analysis, it was not significant in the multivariate model. Similarly, smoking and alcohol consumption showed positive associations in the univariate analysis, but their coefficients in the multivariate model were low (0.32 and 0.41, respectively) and not statistically significant (with respective *p*-values of 0.735 and 0.689). Diabetes also exhibited a positive association in the univariate analysis but was not significant in the multivariate model. These findings highlight that pain (as measured by the VAS) is the most strongly and independently associated factor with increased GAP-43 protein levels, while lifestyle factors—although positively associated in the univariate analysis—did not retain significance in the multivariate context.

To summarize, for the analysis of the potential influence of lifestyle factors on GAP-43 expression levels, both univariate and multivariate regression models were employed. The univariate analyses revealed significant associations between smoking, alcohol consumption, and elevated GAP-43 protein levels; however, the multivariate regression analysis, which accounted for multiple variables simultaneously, demonstrated that these lifestyle factors did not retain independent significance when pain severity was included in the model. Pain severity emerged as the strongest and most consistent predictor of GAP-43 levels, indicating that lifestyle factors may act as confounding variables rather than independent influencers. This suggests that the observed associations between lifestyle behaviors and GAP-43 levels may be mediated by their impact on pain severity.

## 4. Discussion

This study uniquely bridges the gap in knowledge by investigating the changes in expression of GAP-43 within the ligamentum flavum in the context of degenerative changes and pain severity. The findings provide insights into molecular alterations that may underpin pain and functional impairment, positioning GAP-43 as a potential biomarker and therapeutic target in degenerative spinal stenosis. The current study provides critical insights into the role of GAP-43 in the pathophysiology of degenerative lumbosacral spinal stenosis.

The yellow ligamentum flavum was utilized as a comparative material in this study, obtained post-mortem and preserved with RNAlater reagent. This reagent—as specified by the manufacturer—safeguards tissue integrity, making it suitable for transcriptomic and proteomic analyses [25,26]. Notably, for research involving human-derived clinical material in degenerative spinal diseases, post-mortem sampling remains the only feasible approach. Prior studies have corroborated that post-mortem tissue collection is a reliable method, ensuring the validity of comparative analyses, such as those evaluating molecular concentrations in tissues impacted by degenerative changes [27,28].

However, our observation of a discrepancy between mRNA downregulation and protein upregulation warrants further discussion. Additionally, it has been suggested that inflammatory cytokines could mediate such transcriptional downregulation while concurrently enhancing translation via post-transcriptional mechanisms [29,30].

Transcription—the initial step in gene expression—occurs in the nucleus, where RNA polymerase II synthesizes mRNA from a DNA template. The mRNA undergoes maturation and is transported to the cytoplasm for translation. Ribosomes decode the mRNA into a polypeptide chain, which folds into a functional protein [31,32,33,34,35,36]. These processes are regulated independently: transcription by transcription factors and epigenetic changes, and translation by mRNA stability and post-transcriptional mechanisms such as microRNAs [34,35]. The same mRNA can be translated multiple times, amplifying protein levels despite reduced mRNA expression, which may explain the observed GAP-43 protein upregulation [37,38]. Post-translational modifications, including phosphorylation, further stabilize GAP-43 under pathological conditions [39].

The study demonstrated significant alterations in GAP-43 expression in the yellow ligamentum flavum of patients with degenerative spinal stenosis. Our findings align with foundational research by Benowitz and Routtenberg [40], who described GAP-43 as a pivotal molecule in neuronal development and axonal regeneration. This protein has been characterized as a critical mediator of nerve growth and repair, particularly under conditions of injury and regeneration, making its upregulation a hallmark of neuronal plasticity [40]. These studies underline the biological relevance of GAP-43 in adaptive responses to neural injury and degeneration [41].

The observed increase in GAP-43 protein levels in the ligamentum flavum may reflect a compensatory neuroplastic response to ischemic nerve root injury—a key pathological mechanism in lumbar spinal stenosis [20,21]. GAP-43, known for its role in axonal growth, synaptic remodeling, and intracellular calcium signaling, has been shown to play a pivotal role in neuronal repair following ischemic injury [13,14,19,20]. In this context, the elevated levels of GAP-43 in ligamentous tissue could contribute to the modulation of pain pathways through increased neuroplasticity. Specifically, GAP-43 may enhance synaptic remodeling and nerve sprouting in response to nerve root compression, potentially amplifying pain perception and contributing to chronic pain syndromes associated with spinal stenosis [18]. Furthermore, the influence of GAP-43 on intracellular calcium dynamics may play a role in pain sensitization—a process that is crucial in the development of neuropathic pain [14]. These findings suggest that GAP-43 may act as both a marker of ligament hypertrophy and a modulator of pain generation through its involvement in neuroplastic processes linked to ischemic nerve root injury [42].

*GAP-43* mRNA levels were markedly downregulated in the study group compared to the controls, with an inverse correlation to pain severity, showing progressively lower expression as pain intensified. Conversely, the protein levels of GAP-43 were significantly elevated in the study group, with concentrations rising in parallel with increasing pain severity, as confirmed by the results of both ELISA and Western blot analyses. While lifestyle factors (e.g., smoking and alcohol consumption) and comorbidities (e.g., diabetes) were associated with higher GAP-43 protein levels, these factors were not independently significant when adjusted in the multivariate analyses. Pain severity emerged as the strongest and most consistent factor influencing GAP-43 levels, highlighting its potential role as a biomarker of ligamentum flavum degeneration. Our findings highlight the complex relationship between lifestyle factors and GAP-43 expression levels. While univariate analyses demonstrated significant associations between smoking, alcohol consumption, and elevated GAP-43 protein levels, these relationships were not independently significant in the multivariate models. Instead, pain severity was identified as the primary independent predictor of GAP-43 expression levels. This suggests that lifestyle factors such as smoking and alcohol consumption may indirectly influence GAP-43 expression by contributing to increased pain severity. The lack of independent significance of lifestyle factors in multivariate models underscores the importance of considering pain severity as a central factor driving changes in GAP-43 expression. These findings align with previous studies indicating that neuroplasticity-related proteins are primarily modulated by pain and injury, rather than by lifestyle factors alone. Future research should explore whether interventions targeting pain modulation could more effectively reduce GAP-43 expression and improve clinical outcomes in patients with spinal stenosis. This observation complements the findings of Phillips et al., who reported that lifestyle factors exacerbate inflammation and oxidative stress in neural tissues, potentially amplifying degenerative processes [42]. Smoking has long been identified as a significant risk factor for spinal degeneration, primarily due to its promotion of oxidative stress and chronic inflammation [43]. These mechanisms likely contribute to the observed upregulation of GAP-43 protein as a maladaptive response to injury and degeneration [44,45]. The upregulation of GAP-43 in our study aligns with evidence suggesting that the detrimental effects of smoking on spinal health are partly mediated by neuroplasticity-related proteins, which act in response to heightened tissue damage and repair demands [46].

Similarly, alcohol consumption is known to impair hydration of intervertebral disks, reducing their structural integrity and accelerating degeneration [47]. These structural changes could stimulate compensatory upregulation of the GAP-43 protein as part of a response to neural and tissue damage. In addition, alcohol-induced disruptions to dietary and sleep patterns likely contribute to the chronic inflammation observed in degenerative conditions [48,49,50]. Casoli et al. have investigated the effects of alcohol consumption and aging on GAP-43 protein levels in the hippocampus, revealing an age-dependent decline (−47%) in older rats and an ethanol-induced increase (+81%) exclusive to this group. Despite reduced GAP-43 levels with aging, the hippocampus demonstrated a remarkable ability to compensate for alcohol toxicity [51]. In the context of our study, a similar ethanol-associated increase in GAP-43 protein was observed in individuals with a history of alcohol consumption, suggesting a shared compensatory mechanism in response to neural stress or damage. However, while Casoli et al. focused on the hippocampus and aging [51], our findings extend these insights to the ligamentum flavum, emphasizing the role of GAP-43 in maladaptive neuroplasticity linked to degenerative spinal stenosis. The elevated GAP-43 protein levels in alcohol consumers within our study reflect these underlying mechanisms, suggesting that the protein’s role extends to compensatory neuronal plasticity in response to disk and tissue degeneration.

Our findings of higher GAP-43 protein levels in diabetic individuals align with research highlighting diabetes as a risk factor for spinal degeneration [52,53]. Similarly to BDNF, the increased GAP-43 protein levels in diabetics may represent a maladaptive response aimed at mitigating neural injury in the context of progressive tissue degeneration [54,55].

Additionally, obesity—which is frequently associated with diabetes—contributes to metabolic and inflammatory dysregulation, further aggravating spinal degeneration. Studies on neuroplasticity-related proteins, including BDNF, have suggested that inflammation and oxidative stress in obesity promote their upregulation. Our findings of elevated GAP-43 protein levels in overweight and obese individuals suggest a similar pattern, where GAP-43 acts as a mediator of neural responses to the compounding effects of obesity, metabolic imbalance, and mechanical stress [54,55,56].

This study presents several limitations that should be considered when interpreting the results. One significant limitation is its cross-sectional design, which precludes establishing causality between GAP-43 expression and degenerative changes in spinal stenosis. Furthermore, while tissue samples were appropriately collected and processed according to established protocols, variability in sample quality and potential post-mortem changes in the control group tissue cannot be entirely ruled out. These factors may affect the generalizability of the results. The sample size—although adequate for preliminary analyses—may limit the broader applicability of the study findings, and the control group’s composition could introduce confounding variables due to differences in demographic and clinical characteristics. Additionally, histological evaluations and molecular analyses were performed by independent investigators to ensure their reliability, but the lack of GAP-43 detected in immunohistochemical staining, despite its quantification by other methods, raises questions about the sensitivity of the applied technique or potential antigen masking. Further research using advanced imaging techniques and a longitudinal design could provide more comprehensive insights into the dynamics of GAP-43 expression in degenerative spinal conditions.

Despite these limitations, the study’s multifaceted analytical approach, incorporating RT-qPCR, ELISA, and Western blot, strengthens its findings.

## 5. Conclusions

In conclusion, this study highlighted the potential of GAP-43 as a biomarker for pain severity and disease progression in degenerative spinal stenosis, while also emphasizing the influence of lifestyle factors such as smoking and alcohol consumption, as well as comorbid conditions such as diabetes. The observed correlation between GAP-43 protein levels and pain intensity, alongside its association with certain lifestyle behaviors, underscores its practical value in clinical settings. These findings suggest that GAP-43 not only reflects the pathology of the disease, but may also be modulated by external factors, making it a potential target for both diagnostic and therapeutic interventions.

## Figures and Tables

**Table 1:** Concentrations of GAP-43 in the study and control groups, with differences based on VAS pain severity levels (ELISA results).

Group	GAP-43 Concentration [ng/mL]	*p*-Value
Control	0.54 ± 0.87	<0.0001 ^1^
Study	5.57 ± 0.21	
Pain severity (VAS) 4	5.87 ± 0.23	0.032 ^2^ 0.021 ^3^ 0.019 ^4^ 0.017 ^5^ 0.015 ^6^ 0.039 ^7^ 0.026 ^8^ 0.012 ^9^ 0.011 ^10^
Pain severity (VAS) 5	5.71 ± 0.24	
Pain severity (VAS) 6	6.69 ± 0.19	
Pain severity (VAS) 7	6.51 ± 0.19	
Pain severity (VAS) 8	6.42 ± 0.21	
Pain severity (VAS) 9	6.41 ± 0.23	
Pain severity (VAS) 10	6.39 ± 0.28	

**Table 2:** Comparison of *GAP-43* mRNA expression levels in yellow ligamentum flavum based on gender, BMI, diabetes, smoking, and alcohol consumption in study and control groups.

	Control Group		Study Group	
Comparison (Control/Study Group)	mRNA Expression (^2-∆∆Ct^)	*p*-Value of Student’s Two-Tailed *t*-Test ^1^ or ANOVA ^2^	mRNA Expression (^2-∆∆Ct^)	*p*-Value of Student’s Two-Tailed *t*-Test ^1^ or ANOVA ^2^
Gender				
Female (n = 39/n = 46)	2.98	0.54 ^1^	0.61 ± 0.11	0.83 ^1^
Male (n = 46/n = 50)	2.18		0.54 ± 0.12	
BMI (kg/m^2^)				
Normal (n = 32/n = 40)	1.00	0.43 ^2^	0.59 ± 0.09	0.82 ^2^
Overweight (n = 34/n = 32)	1.3		0.62 ± 0.18	
Obesity (n = 19/n = 24)	1.21		0.54 ± 0.23	
Diabetes				
No (n = 44/n = 46)	1.98	0.71 ^1^	0.59 ± 0.31	0.78 ^1^
Yes (n = 41/n = 40)	2.09		0.57 ± 0.31	
Smoking				
No (n = 33/n = 34)	2.54	0.43 ^1^	0.60 ± 0.19	0.89 ^1^
Yes (n = 52/n = 62)	3.50		0.56 ± 0.29	
Drinking Alcohol				
No (n = 12/n = 11)	3.54	0.23 ^1^	0.52 ± 0.19	0.81 ^1^
Yes (n = 73/n = 85)	3.18		0.63 ± 0.09	

BMI, body mass index.

**Table 3:** Comparison of GAP-43 protein expression levels in yellow ligamentum flavum based on gender, BMI, diabetes, smoking, and alcohol consumption in study and control groups.

	Control Group		Study Group	
Comparison (Control/Study Group)	Protein Expression [ng/mL]	*p*-Value of Student’s Two-Tailed *t*-Test ^1^ or ANOVA ^2^	Protein Expression [ng/mL]	*p*-Value of Student’s Two-Tailed *t*-Test ^1^ or ANOVA ^2^
Gender				
Female (n = 39/n = 46)	0.56 ± 0.04	0.93 ^1^	5.61 ± 0.14	0.87 ^1^
Male (n = 46/n = 50)	0.52± 0.08		5.52 ± 0.12	
BMI (kg/m^2^)				
Normal (n = 32/n = 40)	0.61 ± 0.10	0.82 ^2^	5.54 ± 0.28	0.89 ^2^
Overweight (n = 34/n = 32)	0.56 ± 0.08		5.57 ± 0.31	
Obesity (n = 19/n = 24)	0.52 ± 0.09		5.59 ± 0.44	
Diabetes				
No (n = 44/n = 46)	0.52 ± 0.11	0.87 ^1^	3.54 ± 0.35	<0.0001 ^1^
Yes (n = 41/n = 40)	0.55 ± 1.10		7.60 ± 0.98	
Smoking				
No (n = 33/n = 34)	0.61 ± 0.12	0.12 ^1^	4.97 ± 0.43	<0.0001 ^1^
Yes (n = 52/n = 62)	0.54 ± 0.14		6.17 ± 0.19	
Drinking Alcohol				
No (n = 12/n = 11)	0.43± 0.11	0.04 ^1^	5.01 ± 0.19	<0.0001 ^1^
Yes (n = 73/n = 85)	0.65 ± 0.15		6.13± 0.17	

BMI, body mass index. Data are presented as mean ± standard deviation.

**Table 4:** Univariate regression analyses of variables associated with GAP-43 levels in the control and study group for yellow ligamentum flavum.

Characteristic	Expression Level	Control Group		Study Group	
		r	R^2^	r	R^2^
Gender	mRNA	0.19	0.36	0.25	0.06
	Protein	0.22	0.05	0.28	0.08
BMI (kg/m^2^)	mRNA	0.54	0.29	0.68	0.46
	Protein	0.68	0.46	0.81	0.66
Diabetes	mRNA	0.52	0.27	0.76	0.58
	Protein	0.58	0.34	0.72	0.52
Smoking	mRNA	0.18	0.36	0.85	0.72
	Protein	0.8	0.64	0.89	0.79
Drinking Alcohol	mRNA	0.83	0.69	0.91	0.83
	Protein	0.88	0.77	0.96	0.92

BMI, body mass index; r, correlation coefficient.

**Table 5:** Multivariate regression analyses of variables associated with GAP-43 levels in the control and study group for yellow ligamentum flavum.

Characteristic	Expression Level	Control Group		Study Group	
		Coefficient	*p*-Value	Coefficient	*p*-Value
Gender	mRNA	-	0.62	-	0.304
	Protein	-	0.48	-	0.272
BMI (kg/m^2^)	mRNA	0.17	0.28	0.401	<0.0001
	Protein	0.29	0.08	0.385	<0.0001
Diabetes	mRNA	0.16	0.31	0.467	0.026
	Protein	0.25	<0.0001	0.495	0.029
Smoking	mRNA	0.31	0.17	0.332	0.018
	Protein	0.37	<0.0001	0.322	0.015
Drinking Alcohol	mRNA	0.41	0.21	0.345	<0.0001
	Protein	0.43	<0.0001	0.278	<0.0001

BMI, body mass index; r, correlation coefficient.

**Table 6:** Summary of multivariate regression analysis on GAP-43 protein levels in relation to pain and lifestyle factors.

Factor	Association with GAP-43 Protein (Univariate)	*p*-Value (Univariate)	Coefficient in Multivariate Model	*p*-Value (Multivariate)
VAS Pain Score	Positive	<0.001	3.12	<0.001
BMI	Positive	<0.05	-	Not significant
Smoking	Positive	<0.05	0.32	0.735
Alcohol Consumption	Positive	<0.05	0.41	0.689
Diabetes	Positive	<0.05	-	Not significant

## Data Availability

The data used to support the findings of this study are included in the article. The data cannot be shared due to third-party rights and commercial confidentiality.
